# In the eastern Mediterranean, the rat lungworm *Angiostrongylus cantonensis* is absent or extremely rare according to recent surveys

**DOI:** 10.1007/s00436-025-08580-3

**Published:** 2025-11-10

**Authors:** Lucia Anettová, Anna Šipková, Petr Cibulka, Vivienne Velič, Reham Fathey Ali, David Modrý

**Affiliations:** 1 Department of Veterinary Sciences, Faculty of Agrobiology, Food and Natural Resources, Czech University of Life Sciences, Prague, Czech Republic; 2 Department of Botany and Zoology, Faculty of Science, Masaryk University, Brno, Czech Republic; 3 Department of Pathology and Parasitology, Faculty of Veterinary Medicine, University of Veterinary Sciences Brno, Brno, Czech Republic; 4 Department of Zoology and Agricultural Nematology, Faculty of Agriculture, Cairo University, Giza, Egypt; 5 Department of Organic Crop Production, Faculty of Organic Agriculture, Heliopolis University, Cairo, Egypt; 6 Institute of Parasitology, Biology Center of Czech Academy of Sciences, Ceske Budejovice, Czech Republic

**Keywords:** Emerging zoonotic disease, Invasive nematode, Cyprus, Egypt

## Abstract

This study summarizes the findings of a comprehensive field investigation into the presence of *Angiostrongylus cantonensis*, a zoonotic nematode responsible for meningitis, in selected regions of the eastern Mediterranean. Samples were collected from intermediate hosts (gastropods), definitive hosts (rats), and potential paratenic hosts (reptiles) across Cyprus and the northern part of Egypt. Reptile and gastropod samples were analysed using a species-specific and highly sensitive LAMP assay, while rats were dissected and examined for adult nematodes in their pulmonary arteries. All samples from the surveyed localities tested negative for the parasite and its DNA. These findings led the authors to conclude that *A. cantonensis* is either absent or occurs at a very low prevalence in the areas studied in the eastern Mediterranean.

## Introduction

The rat lungworm *Angiostrongylus cantonensis* is a zoonotic nematode causing neurological disease and death in humans, mammals, and birds (Cowie 2013a). Native to southeast Asia, it is now established in the western Mediterranean (Galán-Puchades et al. 2023; Jaume-Ramis et al. 2023; Pandian et al. 2025). This study explored the understudied eastern Mediterranean for *A. cantonensis*, where published data are scarce. In Egypt, past reports (Yousif and Ibrahim 1978; Ibrahim 2007) were based solely on morphology and lacked molecular confirmation. To address these gaps, we conducted systematic surveys in Cyprus and Egypt, screening potential intermediate, paratenic, and definitive hosts. Our goals were to clarify parasite distribution in Cyprus and update Egypt’s current status. The limited reporting of negative findings may, in part, reflect publication bias, as studies with null results are often underrepresented in the scientific literature, despite their important contribution to understanding disease distribution patterns.

## Materials and methods

**Fig. 1:**
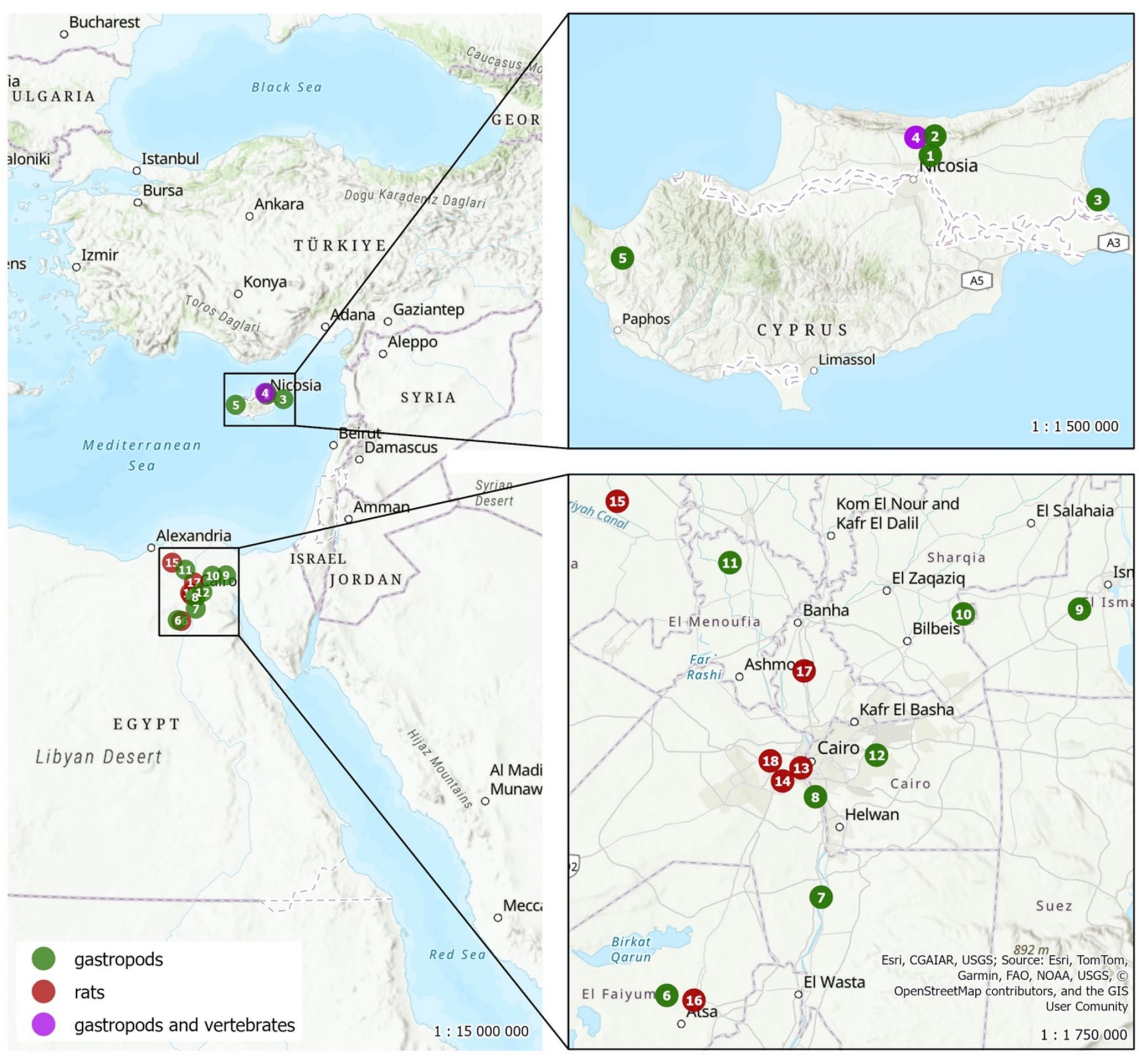
Maps of sampling areas in Cyprus and Egypt for the detection of *A. cantonensis* in various hosts. The maps were created in ArcGIS; base map source: Esri, CGIAR, USGS; data sources: Esri, TomTom, Garmin, FAO, NOAA, USGS, © OpenStreetMap contributors, and the GIS User Community. Note: see Table 1 for all species collected and sample counts

At the Cyprus Wildlife Research Institute (CWRI), the authors examined 10 lizard samples from various species individually, including the Mediterranean house gecko (*Hemidactylus turcicus*), the spotted skink (*Chalcides ocellatus*), and Schneider’s skink (*Eumeces schneideri)*, as reptiles are recognized as potential paratenic hosts of *A. cantonensis* (Anettová et al. 2024, 2025). In cases with multiple specimens of the same species, reptiles were examined in pools, each consisting only of individuals of that species. Four Cyprus rock agamas (*Laudakia cypriaca*) were examined collectively in a single pooled sample. Mediterranean chameleons (*Chamaeleo chamaeleon*) were also tested in pooled samples of five to seven individuals, encompassing 33 specimens. In addition, 47 snakes were examined: large whipsnakes (*Dolichophis jugularis*), Montpellier snakes (*Malpolon insignitus*), and coin snakes (*Hemorrhois nummifer*), organized into four pools of 10 individuals each and one pool of seven (Fig. 1; Table 1). Furthermore, 177 gastropods (possible intermediate hosts), comprising 90 specimens of *Eobania vermiculata*, 25 *Theba pisana*, 2 *Cornu aspersum*, and 40 *Albinaria* sp., were analysed in pooled samples containing five to 10 specimens per pool (Table 1). All gastropods were collected in the northern part of the island: Haspolat lake, Buffavento castle, Famagusta, and CWRI (Fig. 1). Additionally, 20 individuals of *Melanopsis praemorsa* from the southern part of the island at Kremiotis waterfall were examined (Fig. 1; Table 1). Morphological identification of all gastropod specimens was carried out in consultation with a trained malacologist. In all tested reptiles, a small piece of liver (approx. 25 µg in the case of individual samples and approx. 10 µg in the case of pools) was tested for a species-specific LAMP analysis; in the case of gastropods, a small piece of foot tissue was used (Baláž et al. 2023). Liver tissue was chosen, as it has been repeatedly reported as the organ with the highest L3 burden in paratenic hosts (Radomyos et al. 1994; Anettová et al. 2025). All reptile specimens were collected from multiple locations across northern Cyprus and were preserved at the centre over the past two years (2022–2023). The samples were stored in a biobank at −20 °C. During a single visit to CWRI, the authors examined all stored reptile samples preserved over the preceding two years. Eight rats (*Rattus norvegicus*), possible definitive hosts, were captured at the centre using snap traps, then dissected and examined for the presence of adult *A. cantonensis* in the pulmonary arteries.

**Table 1:** Sampling localities with coordinates in Cyprus and Egypt, including the list of species collected and the number of samples examined

No. locality	Latitude	Longitude	Locality name	Species (no. samples)
Cyprus – gastropods and vertebrates				
1	35.233014	33.413393	Haspolat lake	*Eobania vermiculata* (20)
2	35.284331	33.410607	Buffavento castle	*Albinaria* sp. (40)
3	35.114913	33.944792	Famagusta	*Eobania vermiculata* (65), *Theba pisana* (25)
4	35.278835	33.384682	CWRI	*Eobania vermiculata* (5), *Cornu aspersum* (2), *Hemidactylus turcicus* (1), *Chalcides ocellatus* (4), *Eumeces schneideri* (3), *Laudakia cypriaca* (4), *Chamaeleo chamaeleon* (33), *Dolichophis jugularis* (20), *Malpolon insignitus* (10), *Hemorrhois nummifer* (17); *Rattus norvegicus* (8)
5	34.9650577	32.4358034	Kremiotis waterfall	*Melanopsis* sp.* (20)
Egypt – gastropods				
6	29.323928	30.743826	Talat, Faiyum	*Monacha obstruct*a (72)
7	29.630328	31.276754	El Saff, Giza	*Monacha obstructa* (10), Lanistes carinatus* (19), Cleopatra bulimoides* (30),
8	29.935966	31.250418	Abu El Numrus, Giza	*Monacha obstructa* (13)
9	30.517185	32.169905	Abu Suweir El Mahata, Ismailia	*Monacha obstructa* (28), Cleopatra bulimoides* (5), *Oxyloma elegans* (21)
10	30.499198	31.761602	Abu Hammad, Al-Sharqia	Lanistes carinatus* (4), *Bellaymya* sp.* (12), *Eobania vermiculata* (41)
11	30.645667	30.937313	Tala-Toukh Dalka, Monufeia	*Monacha obstructa* (13), *Oxyloma elegans* (20), *Bellamya* sp.* (18), *Eobania vermiculata* (13)
12	30.066776	31.463156	El Banafseg, New Cairo	*Eobania vermiculata* (10)
Egypt – rats				
13	30.023753	31.199614	Boulaq Al Dakrour, Giza	*Rattus norvegicus* (10), *Rattus rattus* (11)
14	29.983468	31.134945	Al Haram, Giza	*Rattus norvegicus* (9), *Rattus rattus* (3)
15	30.82652	30.535063	El Delengat, Beheira	*Rattus norvegicus* (21), *Rattus rattus* (2)
16	29.31000	30.839454	Faiyum	*Rattus norvegicus* (10), *Rattus rattus* (12)
17	30.319408	31.204865	Toukh, Qalyubia	*Rattus norvegicus* (1)
18	30.044536	31.091363	Abou Rawash, Giza	*Rattus norvegicus* (8)

CWRI = Cyprus Wildlife Research Institute. Freshwater gastropods are marked with an asterisk (*). Reptile specimens examined at CWRI were collected across northern Cyprus during 2022–2023 and archived in the CWRI biobank

In Egypt, the presence of *A. cantonensis* was investigated by evaluating potential hosts from 13 localities: Talat (Faiyum), El Saff (Giza), Abu El Numrus (Giza), Abu Suweir El Mahata (Ismailia), Abu Hammad (Al-Sharqia), Tala-Toukh Dalka (Monufeia), El Banafseg (New Cairo), Boulaq Al Dakrour (Giza), Al Haram (Giza), El Delengat (Beheira), Faiyum, Toukh (Qalyubia), Abou Rawash (Giza) (Fig. 1; Table 1). Eighty-seven rats were collected using live traps across six distinct localities (Fig. 1). Based on morphological characteristics, they were identified as *Rattus norvegicus* and *Rattus rattus* (Table 1). The rats were euthanized with T61, and dissections were performed with a focus on the pulmonary arteries and the right ventricle.

Six species of gastropods (329 individuals): *Monacha obstructa* (136), *Lanistest carinatus* (23), *Cleopatra bulimoides* (35), *Oxyloma elegans* (41), *Bellamya* sp. (30), and *Eobania vermiculata* (64), were sampled from seven localities in Egypt (Fig. 1; Table 1). Each gastropod was placed in a separate plastic bag and crushed to facilitate tissue disruption. Tap water was added to the bags, and the samples were incubated overnight to promote the release of larvae into the liquid medium, exploiting the ability of larvae to emerge from dead gastropods as demonstrated by Modrý et al. (2021). Following the incubation period, sediment was carefully collected for further analysis. Microscopic examination was performed to identify the presence of larvae. Additionally, the molecular detection of *A. cantonensis* DNA was conducted using a species-specific LAMP assay (Baláž et al. 2023).

Approximately 150 µL of sediment was used for DNA extraction using PrepMan^®^ (Ultra Sample Preparation Reagent, Thermo Fisher Scientific). The sediment and reagent were mixed in a 1:1 ratio in 2 mL vials. The mixture for DNA extraction was heated at 99 °C in a water bath for 10 min according to the manufacturer’s instructions. Pools of three samples of extracted DNA from sediment were used immediately for LAMP analysis.

## Results and discussion

All samples from potential hosts in Cyprus and Egypt tested negative for *A. cantonensis*, indicating absence or very low prevalence. This aligns with previously published large-scale distribution models that predict thermal and humidity limits to parasite establishment in the eastern Mediterranean (York et al. 2014), where drier conditions and colder winters prevail (Lionello et al. 2006; Harding and Palutikof 2009; Finné et al. 2011).

However, our single-season survey, particularly in Egypt (April–May 2024), and reliance on rodent dissection without molecular confirmation may have missed seasonal prevalence fluctuations or early infections in rodents (Cowie 2013a; Rivory et al. 2025). Future work should employ year‐round sampling with larger host cohorts and integrate both morphological and PCR diagnostics.

Despite no detected parasite circulation, *A. cantonensis* poses a zoonotic risk through contact with infected gastropods or ingestion of intermediate and paratenic hosts, especially given ongoing rat and gastropod movements via global trade and travel (Cowie 2013b; Paredes-Esquivel et al. 2023; Rollins et al. 2023; Šipková et al. 2024). Enhanced surveillance at entry points, targeted ecological studies of introduction pathways, and publication of both positive and negative findings are essential to refine distribution models, inform public health strategies and predict potential spillover in the eastern Mediterranean.

## Data Availability

All data obtained in this study are included in the article.
